# A Metabolic Network Mediating the Cycling of Succinate, a Product of ROS Detoxification into α-Ketoglutarate, an Antioxidant

**DOI:** 10.3390/antiox11030560

**Published:** 2022-03-16

**Authors:** Félix Legendre, Alex MacLean, Sujeenthar Tharmalingam, Vasu D. Appanna

**Affiliations:** 1 School of Natural Sciences, Laurentian University, Sudbury, ON P3E 2C6, Canada; flegendre@laurentian.ca (F.L.); amaclean@laurentian.ca (A.M.); sutharmalingam@nosm.ca (S.T.); 2 Northern Ontario School of Medicine, Laurentian University, Sudbury, ON P3E 2C6, Canada

**Keywords:** sulfur stress, oxidative stress, KG, succinate recycling, metabolic network, glutamate decarboxylase

## Abstract

Sulfur is an essential element for life. However, the soil microbe *Pseudomonas (P.) fluorescens* can survive in a low sulfur environment. When cultured in a sulfur-deficient medium, the bacterium reprograms its metabolic pathways to produce α-ketoglutarate (KG) and regenerate this keto-acid from succinate, a by-product of ROS detoxification. Succinate semialdehyde dehydrogenase (SSADH) and KG decarboxylase (KGDC) work in partnership to synthesize KG. This process is further aided by the increased activity of the enzymes glutamate decarboxylase (GDC) and γ-amino-butyrate transaminase (GABAT). The pool of succinate semialdehyde (SSA) generated is further channeled towards the formation of the antioxidant. Spectrophotometric analyses, HPLC experiments and electrophoretic studies with intact cells and cell-free extracts (CFE) pointed to the metabolites (succinate, SSA, GABA) and enzymes (SSADH, GDC, KGDC) contributing to this KG-forming metabolic machinery. Real-time polymerase chain reaction (RT-qPCR) revealed significant increase in transcripts of such enzymes as SSADH, GDC and KGDC. The findings of this study highlight a novel pathway involving keto-acids in ROS scavenging. The cycling of succinate into KG provides an efficient means of combatting an oxidative environment. Considering the central role of KG in biological processes, this metabolic network may be operative in other living systems.

## 1. Introduction

Sulfur is an essential element for life. It is present in macromolecules and plays a key role in redox processes, in large part due to sulfhydryl groups of proteins. In fact, the interplay between sulfhydryl groups (-SH) and disulfide bonds (S-S) is at the center of the oxidative state of living organisms from bacteria to humans [1,2,3]. Free sulfhydryl moieties are required for the proper functioning of numerous enzymes [4]. Sulfur also participates in the synthesis of Fe-S clusters, which are essential for the activity of enzymes involved in energy metabolism, including the TCA cycle and oxidative phosphorylation. For instance, aconitase, fumarase, and electron transport chain enzymes contain Fe-S [5,6,7]. Abnormal biosynthesis and maintenance of these clusters lead to the accumulation of free cytosolic iron and the creation of free radicals through the Fenton reaction [8,9]. As a result, sulfur starvation contributes to a highly oxidative environment where the TCA cycle and oxidative phosphorylation are severely impeded [10].

The metabolite α-ketoglutarate (KG) is an intermediate of the TCA cycle. Its central position in energy metabolism makes it a pivotal regulatory component in different biological processes, ranging from amino acid homeostasis to genetic modifications as well as cell signaling [5,11,12]. The metabolite KG has been recognized for its ability to combat oxidative stress generated by reactive oxygen species (ROS) [13,14,15,16]. It can readily neutralize ROS with the concomitant formation of succinate, carbon dioxide and water. This attribute makes KG an important protective asset against cellular oxidative stress arising from exposure to hydrogen peroxide to metal stress, metabolic dysfunctions, and a range of other challenges [17,18]. In fact, high concentrations of metals like iron and gallium have been shown to exert toxic effects on biological systems in a manner that perturb Fe-S cluster biosynthesis [8,19]. An increase in NADPH, superoxide dismutase, catalase and glutathione peroxidase coupled with the enhanced synthesis of keto-acids like KG are associated with the core anti-oxidative defense strategies in numerous organisms [18,20].

The soil microbe *Pseudomonas (P.) fluorescens* is a nutrient-versatile bacterium found in diverse environments including soil and water bodies. It can orchestrate tightly regulated metabolic adaptations to grow and thrive under different abiotic challenges such as oxidative, metal and nutrient stress [18,21,22,23]. When grown in a glutamine mineral medium without added sulfur, *P. fluorescens* switches its metabolism towards NADPH production and NADH utilization to better survive in the oxidative environment triggered by sulfur starvation, a stress responsible for the superoxide and H_2_O_2_ [10,24]. This pathway involves using the NAD^+^ pool in the cell to produce NADP^+^ through NAD^+^ kinase. NADP^+^-dependent glutamate dehydrogenase and isocitrate dehydrogenase are upregulated to produce the antioxidant NADPH. In this study, the ability of the organism to reprogram its metabolic networks aimed at the production of the antioxidant KG was investigated. Here, we report that oxidative stress evoked by sulfur starvation triggered a metabolic reconfiguration aimed at enhanced synthesis and regeneration of KG. A novel pathway, reliant on the enzymes succinate semialdehyde dehydrogenase (SSADH) and alpha-ketoglutarate decarboxylase (KGDC), orchestrates the cycling of succinate, a by-product of ROS neutralization into KG, the antioxidant. Glutamate decarboxylase (GDC), fumarate reductase (FRD) and isocitrate lyase (ICL) participate in increased formation of KG. The efficacy of this metabolic adaptation involving the enzymes of the glyoxylate cycle, GABA shunt and the TCA directed towards intracellular oxidative environment provoked by sulfur stress is also discussed. These findings further highlight the significance of metabolism and keto-acids in modulating ROS.

## 2. Material and Methods

### 2.1. Bacterial Cultures and Biomass Measurement

*Pseudomonas fluorescens* 13525 was obtained from the American Type Culture Collection (Manassas, VA, USA). It was cultured in a defined mineral medium with glutamine (19 mM) as the only source of carbon. The control medium contained 42 mM Na_2_HPO_4_, 22 mM KH_2_PO_4_ and 0.8 mM of MgSO_4_·7H_2_O. In the stressed medium MgSO_4_·7H_2_O, was substituted by MgCl_2_ (0.8 mM). Trace metals were added to the culture as previously described in [25]. Media was autoclaved for 20 min at 121 °C. The cultures had a volume of 200 mL and were inoculated with 1 mL of *P. fluorescens* preculture as previously described in [25] and grown on a Gyrotory^®^ Water Bath Shaker Model G76 (New Brunswick Scientific, Midland, ON, Canada) at ambient temperature. Biomass measurements were taken using the Bradford assay [26] to quantify the amount of soluble protein as the indicator of cellular biomass. Ten milliliters (mL) of microbial culture were spun down at 12,000× *g* for 20 min. The pellet containing the bacterial cells was resuspended in 1 mL of 0.5M NaOH and soluble proteins were assayed in triplicate.

### 2.2. Cell Fractionation and Metabolic Profiling

The pellets harvested after centrifugation were washed with approximately 100 mL of 0.85% m/v sodium chloride and the samples were centrifuged at 12,000× *g* for 20 min. The pellet containing whole cells was resuspended in 1 mL of Cell Storage Buffer (CSB) (50 mM Tris-HCl, 1 mM phenylmethylsulfonyl fluoride, 1 mM dithiothreitol, pH 7.6) and disrupted using an ultrasonic processor (Johns Scientific, Toronto, ON, Canada). Cellular membrane fraction (mCFE, outer and inner membrane) and soluble fraction (sCFE) were obtained by centrifugation at 110,000× *g* for 3 h at 4 °C. The mCFE was resuspended in 1 mL of CSB and sCFE were stored at 4 °C for a maximum of 7 days and were used for follow-up experimentation.

The HPLC analysis was performed on a Waters 2695 Separation Module HPLC system with a Synergi 4 μm Hydro-RP 80A column (250 × 4.6 mm, San Francisco, CA, USA). The flow rate utilized was 0.2 mL/min. The mobile phase consisted of 5% (*v/v*) acetonitrile, 20 mM KH_2_PO_4_ diluted in Milli-Q water at a pH 2.9 at ambient temperature. Detection of metabolites was done using a Waters 2478 Dual λ absorbance detector, set at a wavelength of 210 nm to detect organic acids. Samples were prepared and run in the HPLC as described previously [18]. Metabolite amounts were determined from the area under the curve of the corresponding peaks using the Empower software (Milford, MA, USA) and identified by comparing their elution time to known standard solutions. The peaks were confirmed by spiking the samples with the appropriate standards and keto-acids were further confirmed by the 2,4-dihydrophenylhydrazine (DNPH) assay [21,27,28]. All enzymatic assays with the intact cells, and cell-free extracts were performed with cells obtained at a similar growth phase (late logarithmic phase) in control and sulfur-deficient media.

### 2.3. Detection of Enzymatic Activity

#### 2.3.1. Blue Native Polyacrylamide Gel Electrophoresis (BN-PAGE) and Enzyme Activity

Activity of specific enzymes was detected by Blue Native Polyacrylamide Gel Electrophoresis (BN-PAGE). A 4–16% acrylamide linear gradient native gel was prepared (50 mM є-aminocaproic acid, 15 mM Bis–Tris, pH 7.0, 4 °C). The gel was cast using the MiniProteanTM2 gel system (Bio-Rad Laboratories, Mississauga, ON, Canada). A spacer of 1.0 mm was used to prepare the gel. A quantity of 60 μg of proteins was loaded into gel wells. The gel chamber was filled with anode buffer (50 mM Bis–Tris, pH 7.0, 4 °C), whereas the space between gels was filled with blue cathode buffer (50 mM Tricine, 15 mM Bis–Tris, 0.2 g/L Coomassie Blue G250, pH 7.0, 4 °C. Membrane protein samples contained 1% (m/V) η-dodecyl β-D-maltoside to help proteins solubilize. Electrophoresis was carried out at a voltage of 75 V and a current of 15 mA until the proteins entered the resolving gel as described in [29,30,31]. Enzymatic activity was detected using the formazan precipitation method, where the gel slices are incubated in a reaction buffer (25 mM Tris-HCl, 5 mM MgCl_2_, pH 7.4) with appropriate substrates and redox markers. The compounds iodonitrotetrazolium (INT) in reduced form produces the formazan precipitate. Phenazine methosulfate (PMS) and dichlorophenolindophenol (DCPIP) are used as electron transfer intermediates to allow the reduction of INT. The concentration of INT and DCPIP/PMS was 0.4 mg/mL for all reactions.

The activity of fumarase (FUM) was probed using a mixture of 5 mM fumarate, 0.5 mM nicotinamide adenine dinucleotide (NAD^+^) and 2 units of malate dehydrogenase (MDH) per lane. Fumarate reductase (FRD) activity was monitored in the membrane fraction using 5 mM fumarate and 0.5 mM nicotinamide adenine dinucleotide reduced form (NADH). Complex I activity was assessed on the membrane fraction using 0.5 mM NADH, without PMS, while Complex IV activity was visualized with 5 mg/mL diaminobenzidine, 13.3 mM of sucrose and 10 mg/mL of cytochrome C. α-ketoglutarate dehydrogenase (KGDH) was tested by using 5 mM α-ketoglutarate, 0.5 mM NAD^+^ and 1 mM CoA. α-ketoglutarate decarboxylase activity was assessed as previously described [32]. Enzymatic activity of alanine transaminase was detected by using a mixture of 5 mM alanine, 5 mM α-ketoglutarate, 0.5 mM NAD^+^ and 5 units of glutamate dehydrogenase. Enzymatic activity of isocitrate lyase was detected by using 5 mM isocitrate, 0.5 mM NAD^+^ and 5 units of lactate dehydrogenase. Succinate semialdehyde enzymatic reaction was observed with 5 mM succinate and 0.5 mM NADH.

#### 2.3.2. Detection of Enzymatic Activity by HPLC and Spectrophotometric Assays

Detection of enzymatic activity was also performed by using HPLC and colorimetric assays. Cell free extract (200 μg) from control and stressed cultures were incubated with substrates of interest and reacted for 30–120 min. Following inactivation of the reactions by heat, the products/reactants were monitored. The activity of glutamate decarboxylase (GDC)was determined by monitoring GABA with glutamate as the substrate. The activity of GABA transaminase (GABAT) was detected by incubating 200 μg protein equivalent of cell free extract with 5 mM of GABA and 5 mM of pyruvate. In this instance, alanine and succinate semialdehyde (SSA) were monitored. The activity of fumarate reductase was tested by incubating 200 μg of protein from the cell free extracts of control and stressed cultures with 2 mM fumarate and 0.2 mM of NADH. The activity of succinate semialdehyde dehydrogenase was assessed using 2 mM succinate and 0.2 mM NAD whereas the activity of α-ketoglutarate dehydrogenase (KGDH) was assessed by using 2 mM KG and 0.2 mM NAD^+^. The absorbance of NADH at 340 nm was monitored by UV-Vis spectrophotometer for 60 s. Relative activity was calculated by dividing the rate of NADH consumption or production of the stressed cell free extract reaction by the rate of the control. The control activity was arbitrarily set to 1. To further confirm the nature of the enzymes, activity bands were excised and incubated in appropriate substrates. Product peaks were monitored by HPLC and by the DNPH assay. Intact (whole) cells were utilized to evaluate the metabolic networks enabling *P. fluorescens* to adapt to sulfur starvation. Four milligrams of these cells isolated from control and stressed cultures were incubated with 10 mM glutamate and the conversion of glutamate to GABA was monitored by incubation of the intact cells with the aid of HPLC. Four milligrams of these cells isolated from control and stressed cultures were incubated with 10 mM succinate and 10 mM sodium bicarbonate resulting in the formation of KG and SSA.

#### 2.3.3. Gene Expression Profiling Using Quantitative Real Time Polymerase Chain Reaction (qRT-PCR)

Total RNA from control and stressed cells was extracted using TRIzol Reagent following the provider’s instructions. Samples were treated with DNAse I to avoid genomic DNA contamination. The reverse transcription reaction as well as qRT-PCR reaction were carried out as previously described [10,32]. Primer sequences were designed using Primer-BLAST and NCBI genome databases. Forward/reverse primer pairs for selected genes were subjected to a validation test by plotting Ct values against cDNA serial dilution concentrations. To be considered acceptable for qRT-PCR experiments, primer pairs had to show a R^2^ value > 0.99 and an efficiency between 90% and 110%. The efficiency was calculated using the formula: efficiency = 10(−1/slope) − 1. The housekeeping genes used to normalize the samples were rpoA and cpn60. The relative mRNA transcript level of each gene was shown as fold change using the ΔΔCT method described in [33] as mRNA fold change where the low sulfur mRNA level is compared to the control mRNA level. The list of primers used can be found in Table 1. Accession numbers were obtained from the NCBI database for *Pseudomonas fluorescens* Pf0-(NC_007492.2) or *Pseudomonas fluorescens* strain ATCC 13525 (NZ_LT907842.1).

### 2.4. Statistical Analysis

All experiments were performed in triplicate with at least three biological replicates. Statistical analysis was performed using Student’s *t*-test (paired; two-tail) on Microsoft Excel, from Microsoft Office 365 (Seattle, WA, USA).

## 3. Results

### 3.1. Oxidative Environment Evoked by Lack of Sulfur Leads to Succinate Accumulation in P. fluorescens

**Figure 1:**
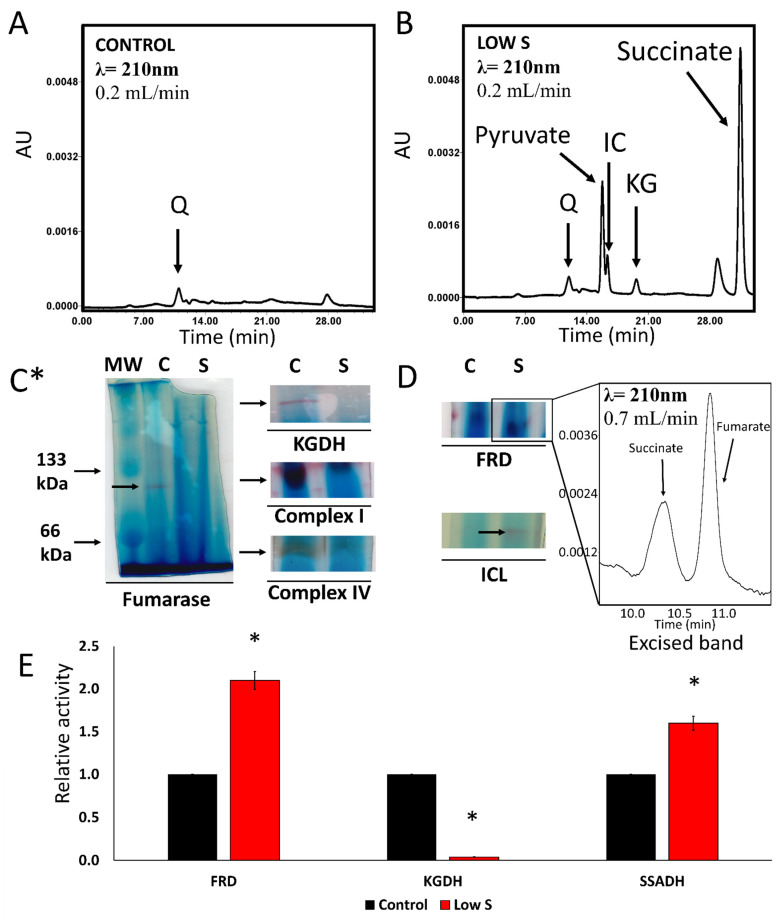
Oxidative stressed provoked by sulfur starvation leads to succinate accumulation in *P. fluorescens*. (**A**). HPLC chromatogram of a spent fluid sample from *P. fluorescens* grown in control conditions. (**B**). HPLC chromatogram of a spent fluid sample from *P. fluorescens* grown in media with no added sulfur. The X axis shows retention time in minutes and the y axis shows absorbance at 210 nm in arbitrary units (AU). (C*). Enzymatic activity analysis of TCA cycle and oxidative phosphorylation enzymes by Blue Native polyacrylamide gel electrophoresis (BN-PAGE). Fumarase, α-ketoglutarate dehydrogenase (KGDH) and (Complex I-IV) activities are decreased in the stressed cells. (**D**). Enzymatic activity analysis of fumarate reductase (FRD) and isocitrate lyase (ICL). FRD and ICL activities are increased under sulfur starvation. The nature of FRD was confirmed by cutting the band and incubating it in reaction buffer with 2 mM fumarate and 0.2 mM NADH. Detection of succinate was done by running the reaction mixture in the HPLC after 24 h. C = Control, S = Low sulfur. (**E**). Enzymatic assays of select enzymes with appropriate substrates by measuring the absorbance of NADH at 340 nm. The rate of reaction was calculated using the slope of absorbance change per minute. The control sample was normalized to 1 and the stressed rate was compared to it, data is shown as relative activity. (Q = glutamine, IC = isocitrate, KG = α -ketoglutarate) Data are represented as means ± standard deviation and is representative of three (3) distinct experiments. n = 3, * = *p* < 0.05.

Although there was no significant change in the growth profile of the microbe grown in control and sulfur-deficient media, a marked difference was observed in the metabolic processes occurring under these conditions. The spent fluid obtained from the sulfur deficient cultures revealed prominent peaks attributable to succinate, pyruvate, isocitrate and KG by HPLC analyses. These peaks were barely discernable in the control cultures (Figure 1A,B). This elevated amount of the dicarboxylic acid, a known product of ROS neutralization characteristic of KG, prompted the evaluation of select enzymes responsible for the TCA cycle and oxidative phosphorylation that are known to be perturbed by oxidative stress in this instance triggered by sulfur starvation. The BN-PAGE analysis of enzymatic activity revealed that fumarase (FUM), KGDH, and complexes I and IV, all sulfur dependent proteins, had decreased activity (Figure 1C*). Inversely, fumarate reductase (FRD), that converts fumarate to succinate, and isocitrate lyase (ICL) that mediates the transformation of isocitrate to succinate and glyoxylate were upregulated in the stressed cultures. The excised band responsible of FRD activity readily generated succinate upon incubation with fumarate and NADH (Figure 1D). Spectrometric studies of select enzymes further confirmed the increased activity of FRD and diminished activity of KGDH in the stressed cells (Figure 1E).

### 3.2. Metabolite and Enzyme Profiles in Cell-Free Extracts (CFEs) from Control and S-Deficient Cultures

**Figure 2:**
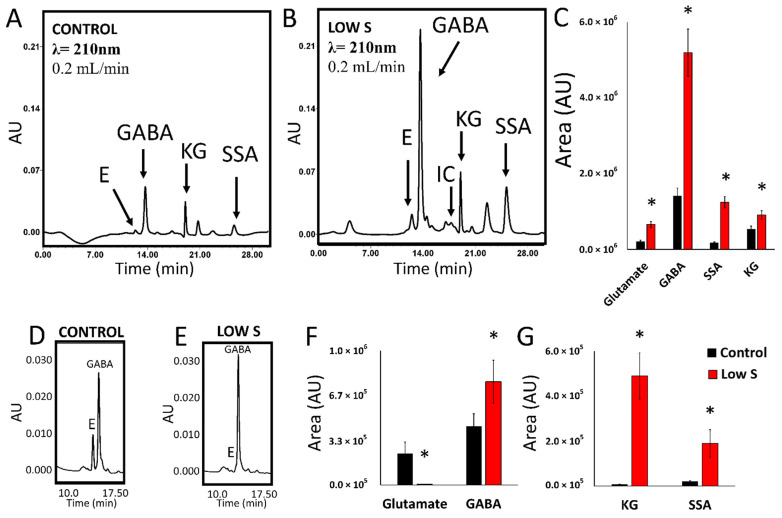
Metabolite profiling in *P. fluorescens* cell free extract and intact cells in control and sulfur-deficient conditions. (**A**). HPLC chromatogram of a cell free extract from *P. fluorescens* grown in control conditions. (**B**). HPLC chromatogram of a cell free extract sample from *P. fluorescens* grown in a media with no added sulfur. Peaks corresponding to glutamate (**E**), isocitrate (IC), γ-aminobutyric acid (GABA), α-ketoglutarate (KG) and succinate semialdehyde (SSA) are shown. (**C**). Quantification of select metabolites in cell free extract samples of *P. fluorescens* grown in control and sulfur-deficient (Low S) conditions. Enrichment in GABA, SSA and KG is seen in sample from sulfur-deficient grown cells. (**D**,**E**). HPLC chromatogram of whole cells *P. fluorescens* control (**D**) and low sulfur (**E**) cells incubated with 10 mM glutamate for two hours. (**F**). Quantification of metabolites in whole *P. fluorescens* cells grown in control and sulfur-deficient (Low S) conditions fed with 5 mM glutamate and incubated for two hours. (**G**). Quantification of metabolites in whole *P. fluorescens* cells grown in control and sulfur-limited (Low S) conditions fed with 10 mM succinate, 10 mM HCO_3_^−^ and incubated for two hours. Succinate metabolism leads to enrichment in KG. Data are represented as means ± standard deviation. n = 3, * = *p* < 0.05.

To further assess the nature of the metabolic shift resulting in the elevated presence of succinate in the spent fluid and probe the diminished activity of enzymes involved in aerobic energy formation, the metabolite and the enzymatic profiles of the CFEs were examined. The HPLC chromatogram of soluble CFEs of *P. fluorescens* cells grown under a lack of S consistently showed higher amounts of KG, γ-aminobutyric acid (GABA) and succinate semialdehyde (SSA) (Figure 2A–C). Additionally, peaks attributable to moieties like isocitrate that were evident in the stressed cells were barely discernable in the controls (Figure 2C). The prominence of GABA in the soluble CFE led us to investigate the ability of the intact cells to consume glutamate and generate GABA. The HPLC analysis showed that *P. fluorescens* harvested from the sulfur-limited culture produced more GABA than the controls when incubated in the presence of glutamate (Figure 2D–F). As succinate was a prominent metabolite in the spent fluid, it was important to decipher if the intact cells were capable of producing KG upon incubation with this dicarboxylic acid and HCO_3_^−^. Indeed, SSA and KG were detected. The former was readily carboxylated to KG. These peaks were not evident in the control cells (Figure 2G).

**Figure 3:**
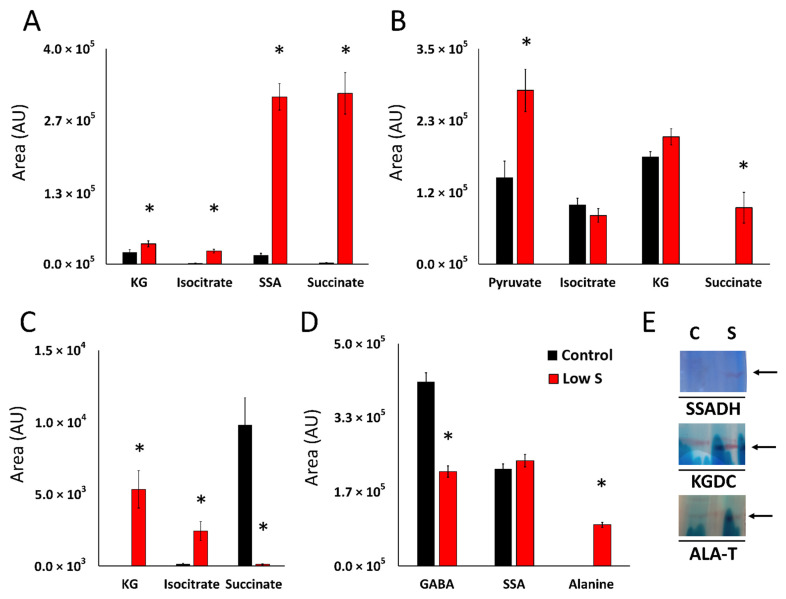
Multiple routes to α-ketoglutarate in *P. fluorescens* subject to sulfur starvation. (**A**). Quantification of metabolites in membrane cell free extracts of *P. fluorescens* grown in control and limited-sulfur (Low S) conditions incubated with 5 mM glutamine for 60 min. Glutamine utilization leads to enrichment of KG, Isocitrate, SSA and succinate. (**B**). Quantification of metabolites in cell free extracts of *P. fluorescens* grown in control (black) and sulfur-limited (red) conditions incubated with 5 mM isocitrate for 60 min. Isocitrate metabolism leads to enrichment of KG, succinate and pyruvate. (**C**). Quantification of metabolites in cell free extracts of *P. fluorescens* grown in control and sulfur-limited (Low S) conditions incubated with 5 mM succinate, 10 mM HCO_3_^−^ for 60 min. Succinate metabolism results in the production of isocitrate and KG. (**D**). Quantification of metabolites in membrane cell free extracts of *P. fluorescens* grown in control and sulfur-deficient (Low S) conditions incubated with 5 mM GABA, 5 mM pyruvate for 60 min. Production of alanine, a by-product of GABAT is identified. (**E**). Activity analysis by BN-PAGE of succinate semialdehyde dehydrogenase (SSADH), α-ketoglutarate decarboxylase (KGDC) and alanine transaminase (ALA-T). SSADH, KGDC and ALA-T. C = control, S = low sulfur. Data are represented as means ± standard deviation. n = 3, * = *p* < 0.05.

Experiments were performed with cell-free extracts in the presence of glutamine, isocitrate and succinate to further assess the nature of the metabolic network resulting in the cycling of succinate into KG. The metabolism of glutamine in the soluble CFE yielded increased amounts of succinate, SSA, KG and isocitrate in the stressed cells. Incubation in the presence of isocitrate gave peaks reflective of pyruvate, KG and succinate. In the presence of succinate and HCO_3_, the sCFE extracts generated KG and isocitrate (Figure 3A–C). Membrane mCFE isolated from the stressed cells readily produced alanine and SSA in higher amounts compared to the control mCFE when incubated with GABA and pyruvate. This pointed to an increased activity of GABA transaminase (GABAT) in the cell free extracts from the culture grown without added sulfur (Figure 3D). To further understand the biochemical networks resulting in the elevated amounts of KG, pyruvate and SSA in the stressed cells, select enzymatic activities were assessed by BN-PAGE. In the stressed cells, KGDC, an enzyme involved in the carboxylation of SSA to KG, was increased concomitant with the augmentation of SSADH activity, an enzyme modulating the synthesis of SSA. As alanine transaminase (ALA-T) can be a source of elevated pyruvate, a precursor to KG, this enzyme was also analyzed by BN-PAGE. A noticeable increase was observed in the stressed cells (Figure 3E). Addition of stressed cells into a control medium readily reversed this biochemical trend (personal observation).

### 3.3. Gene Expression Profiling in P. fluorescens under Sulfur Starvation

**Figure 4:**
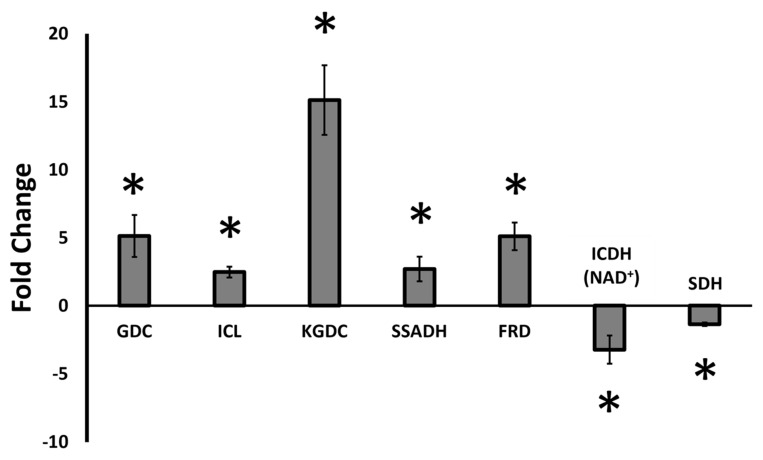
Gene expression profiling of genes involved in KG regeneration under sulfur starvation in *Pseudomonas fluorescens*. The qRT-PCR experiments were performed using primer pairs from Table 1. Relative expression of mRNA transcripts in comparison to control conditions in cells at the late exponential phase was determined using the ΔΔCt method. GDC = glutamate decarboxylase, ICL = isocitrate lyase, KGDC = α-ketoglutarate decarboxylase, SSADH = succinate semialdehyde dehydrogenase, FRD = fumarate reductase = ICDH (NAD^+^) = NAD^+^-dependent isocitrate dehydrogenase, SDH = succinate dehydrogenase. n = 4, * = *p* < 0.05.

Gene expression profiling studies were performed to evaluate if the enzymes in the cycling of succinate to KG observed under sulfur starvation were upregulated during transcription. Quantitative real time polymerase chain reaction (qRT-PCR) experiments revealed that genes responsible for glutamate decarboxylase (GDC), isocitrate lyase (ICL), α-ketoglutarate decarboxylase (KGDC), succinate semialdehyde dehydrogenase (SSADH) and fumarate reductase (FRD) were indeed upregulated significantly in cells from cultures grown in sulfur-deficient media, whereas diminished expression of genes resulting in the enzymes isocitrate dehydrogenase (ICDH-NAD^+^) and succinate dehydrogenase (SDH) were observed. A 15-fold increase KGDC transcript was observed while mRNA responsible for SDH was decreased 2-fold (Figure 4).

## 4. Discussion

The data in this report demonstrate the ability of *P. fluorescens* to survive a sulfur deficient medium by reprogramming its metabolism aimed at the cycling of succinate to KG, an antioxidant. Succinate generated because of ROS detoxification by KG is converted into SSA and subsequently into KG. This provides an elegant cycle to scavenge ROS without an onerous burden on the organism. Sulfur deprivation is known to impose oxidative stress in all organisms, as sulfur is an important component of the redox potential living systems must maintain to live in an aerobic environment. We have recently shown that in a medium with glutamine as the sole carbon source, this microbe elaborates a metabolic network to produce enhanced NADPH and consumption of NADH [10,34]. In this instance, the oxidation carboxylation of KG by ICDH leads to the synthesis of isocitrate and the utilization of NADH. The tricarboxylic acid subsequently supplies NADPH, a process mediated by the enzyme ICDH-NADP^+^ dependent. This strategy of antioxidative defense is complemented with the increased production of KG, an antioxidant. Keto-acids are excellent neutralizers of reactive oxygen and nitrogen species (RONS) [13,35]. While KG produces succinate upon detoxification of RONS, pyruvate is decomposed to acetate following decarboxylation triggered by oxidative stress. Glyoxylate decomposes RONS to release formate [36,37]. These keto-acids are widely utilized as antioxidants in vivo and in vitro in medications and nutrient supplements [38,39,40].

The TCA cycle intermediate KG is a molecule with known antioxidative properties that have been studied from bacteria to humans [1,2,3,17,32,41]. However, the metabolic routes to KG in an environment where sulfur is limiting has not been well-characterized. The involvement of KG in mitigating the oxidative stress evoked by sulfur starvation is apparent from the fact that a significant amount of succinate is found in the spent media of the cultures grown in the absence of added sulfur (Figure 1B). The metabolite KG can readily detoxify ROS with the generation of succinate, a moiety that is also known to act as a signaling metabolite [42]. In fact, aluminum toxicity resulting from the disruption of Fe-S cluster triggers modulation of the TCA cycle and enhanced synthesis of KG and succinate, an observation that has also been reported during oxidative stress and sulfur starvation [18,35,43,44,45]. Not only key enzymes of the TCA cycle are affected but the electron transport chain is also severely perturbed as revealed by the marked inhibition of complexes I and IV (Figure 1). This metabolic shift promotes the utilization of KG in ROS detoxification and reduction in ROS formation because of diminished oxidative phosphorylation. Thus, sulfur starvation, metal toxicity and elevated amounts of H_2_O_2_, all known to increase oxidative tension elicit the reconfiguration of cellular metabolism [10,21,36,43]. The utilization of glutamine, as the only source of carbon and nitrogen in the present study, permits the further exploration of the homeostasis of the antioxidant KG, as this amino acid has to transit via the keto-acid to be further processed in any organism [35,36].

**Figure 5:**
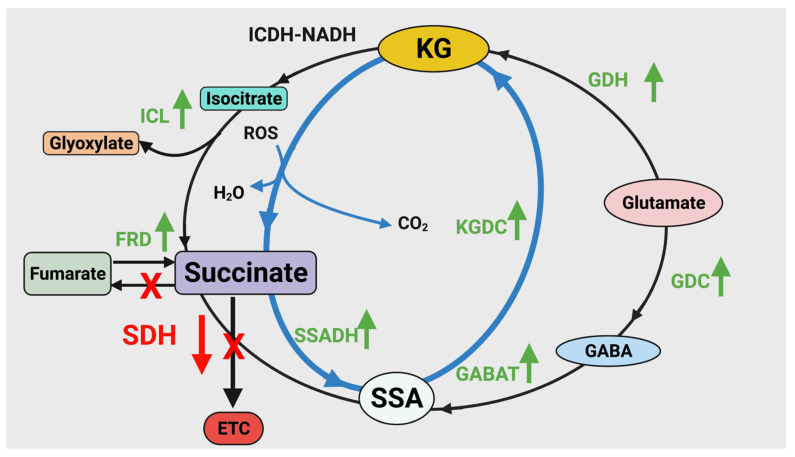
Schematic representation of the metabolic pathway used by *P. fluorescens* to combat sulfur starvation. The dicarboxylic acid is regenerated into KG by SSADH and KGDC. The enzymes ICL and FRD contribute to the succinate pool of succinate while GDC and GABAT maintain SSA homeostasis. ICL = isocitrate lyase, FRD = fumarate reductase, SSADH = succinate semialdehyde dehydrogenase, SDH = succinate dehydrogenase, KGDC = α-ketoglutarate decarboxylase, GABAT = GABA transaminase, GDC = glutamate decarboxylase, GDH = glutamate dehydrogenase, KG = α- ketoglutarate, SSA = succinate semialdehyde. 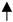 = Cycle producing KG from glutamate, 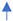 = Regeneration of KG from succinate, the product of ROS detoxification.

Metabolite profiling of *P. fluorescens* under sulfur-limited conditions showed a higher amount of GABA, SSA and KG compared to the controls (Figure 2). This metabolite fingerprint points to a prominent role of GABA shunt in the sulfur-deficient cells. This metabolic network is known to be responsible for various brain functions in higher organisms while in prokaryotes it acts a compatible solute and helps regulates pH [24,46,47]. The enzymes GABA transaminase (GABAT) and glutamate decarboxylase (GDC) are key contributors to this metabolic module and showed increased activity in sulfur-stressed cells (Figure 3C–F). Although this pathway does not directly result in KG production, it generates SSA, a moiety that can be converted to KG with the assistance of KGDC. The activity and expression of this enzyme was markedly elevated. In fact, a 15-fold increase in the transcript was observed (Figure 3G and Figure 4). The increased activity and expression of isocitrate lyase (ICL) and fumarate reductase (FRD) may help supplement the pool of succinate, a metabolite that reacts with NADH to generate SSA with the aid of SSADH, an enzyme that was upregulated more than 2-fold. The significance of the enzyme in the enhanced production of KG in glycerol medium supplemented by micromolar amounts of manganese (Mn) was recently demonstrated [31]. In the present study where sulfur starvation resulting in oxidative stress compels the microbe to augment its KG production. The latter is then utilized in the elimination of ROS leading to the formation of succinate. Succinate is then channeled towards KG synthesis, a process further promoted by the downregulation of SDH, an enzyme reliant on sulfur and the electron transport chain. Increased production of KG orchestrated by glutamate dehydrogenase and ICDH-NADP^+^ has also been shown in *P. fluorescens* in a glutamine culture exposed to H_2_O_2_ [24]. Succinate generated by ICL, FRD and following the ROS-mediated decomposition of KG is routed by SSADH to SSA. The latter is further supplemented by the transamination of GABA and is subsequently transformed into the antioxidant KG. This metabolic reprogramming provides an elegant and potent antioxidative strategy to quell the oxidative environment triggered by sulfur starvation in *P. fluorescens*. The pool of succinate is replenished by isocitrate lyase and fumarate reductase that are known to participate in disparate stress responses in numerous microbial systems [4,42]. The scheme shown in Figure 5 illustrates the metabolic reprogramming occurring in *P. fluorescens* to transform succinate into KG, an antioxidant aimed at the oxidative tension evoked by sulfur starvation.

## 5. Conclusions

In conclusion, *P. fluorescens* invokes a major metabolic reprogramming to combat sulfur starvation-induced oxidative stress. The metabolite KG is the potent antioxidant utilized to detoxify ROS in a medium where glutamine is the sole source of carbon and nitrogen. The pooling and channeling of succinate to SSA provides an efficient route to KG that can be regenerated. The malleability of metabolic pathways and the ability of organisms to use different enzymatic systems usually dedicated to diverse physiological functions represent a unique strategy to fend off abiotic insults. In this instance, the glyoxylate and GABA shunts coupled with components of the TCA cycle enable *P. fluorescens* to neutralize the toxicity associated with a sulfur-limited environment. The fluxes in the concentrations of isocitrate, succinate, GABA, succinate semialdehyde and fumarate are the main effectors fueling the formation of KG, a metabolite in high demand under sulfur starvation. This further confirms that phenotypic changes mediated by metabolites are central to the physiological adaptations most organisms undergo.

## Figures and Tables

**Table 1:** List of primers used for the qRT-PCR analysis.

Gene Name	Enzyme Symbol	Genome ID (Gene Location)	Forward Primer [5′ to 3′]	Reverse Primer [5′ to 3′]
Succinate dehydrogenase (sdhB)	**SDH**	NZ_LT907842.1 (4306753–4307457)	CTCGACGGTCTGTACGAGTG	CTGGACCCAGGAACTTGTCC
Fumarate reductase (sdhA)	**FRD**	NZ_LT907842.1 (2366315–2368087)	GATCGCGACGACGAAAACTG	GGAACTGTCTTCGGCGAGAA
Glutamate Decarboxylase (Gldc)	**GDC**	NZ_LT907842.1 (5096095 5096823)	TTTCGACCTGCCGGAAATGA	TTCACCTCATGGGCCTGTTC
Alpha-ketoglutarate decarboxylase (alkB)	**KGDC**	NZ_LT907842.1 (3673836–3674501)	CCCCAGAAGATCGCCTTGTT	TCGGCATCACCCCATGAAAA
Isocitrate dehydrogenase (NAD^+^) (AceK)	**ICDH (NAD^+^)**	NZ_LT907842.1 (4980207–4981922)	CCCGAGGATGAGATGGCTTC	AAGGCGGGAATTCTTCAGGG
Succinate semialdehyde dehydrogenase (SSADH)	**SSADH**	NC_007492.2 (225316–226758)	GTCGTCAGCTGATGTCGGAA	GTCGAACACGATGAATGGCG
Isocitrate lyase (AceA)	**ICL**	NZ_LT907842.1 (6193725–6195050)	CCCACGGGTCTTCTTTCAGG	ACAAAGTCGGCTACTGGTCG
**Housekeeping Gene:** Chaperonin 60 (cpn60)	**N/A**	NZ_LT907842.1 (5505175–5506821)	GCGACATGATCGAAATGGGC	GCCAGTCGAGCCTTCTTTCT
**Housekeeping Gene:** DNA-directed RNA polymerase subunit alpha (rpoA)	**N/A**	NZ_LT907842.1 (6028685–6029686)	TCCTGTTGTCCTCAATGCCC	TGCAGCTTGATAGCCAGACC

## Data Availability

Data is contained within the article.
